# Aortocaval compression resulting in sudden loss of consciousness and severe bradycardia and hypotension during cesarean section in a patient with subvalvular aortic stenosis

**DOI:** 10.1186/s12871-019-0791-x

**Published:** 2019-07-04

**Authors:** Shouming Chen, Lan Wu, Xiaoqin Jiang

**Affiliations:** 1 Department of anesthesiology, West China Second University Hospital, Sichuan University, No. 20, Section 3, South Renmin Road, Chengdu, 610041 Sichuan province China; 2 Key Laboratory of Birth Defects and Related Diseases of Women and Children (Sichuan University), Ministry of Education, The people of south road 20, Chengdu, Sichuan Province China

**Keywords:** Subvalvular aortic stenosis, Pregnancy, Cesarean section, Hypotension

## Abstract

**Background:**

Maternal cardiac arrest during cesarean section (CS) is an extremely rare but devastating complication. Preventing emergency events from developing into maternal cardiac arrest is one of the most challenging clinical scenarios.

**Case presentation:**

A 35-year-old pregnant woman with subvalvular aortic stenosis who was scheduled for elective CS under epidural anesthesia, and experienced devastating supine hypotensive syndrome, but was successfully resuscitated after delivery.

**Conclusions:**

The performance of tilt position strictly or high-quality continue manual left uterine displacement (LUD) should be performed until the fetus is delivered, otherwise timely delivery of the fetus may be the best way to optimize the deadly condition.

## Background

Subvalvular aortic stenosis (SAS) is one of the common adult congenital heart diseases (CHD), and accounts for 8–20% of all forms of left ventricular outflow tract (LVOT) obstruction [1]. Pregnancy associated with cardiac disease is one of the top three causes of maternal mortality, which incidence is increasing. There have been multiple reports of perimortem cesarean delivery (PMCD) as a treatment option for the mother who has not achieved spontaneous circulation after cardiac arrest due to aortocaval compression [2]. In this report, we presented a case about timely delivery of the fetus preventing maternal cardiac arrest.

## Case presentation

A 35 years old (height, 159 cm; weight, 85 kg; gravida 3, para 1) pregnant woman with SAS presented to our hospital and was scheduled to undergo elective CS under epidural anesthesia at 36 weeks of gestation. An echocardiographic examination at 34 weeks of gestation revealed that the diameter of the beginning portion of the aorta (under the aortic valve) was narrowed by about 17 mm. The diameter of the ascending aorta was normal, the average pressure gradient was 40 mmHg, and the ejection fraction was 67%.

In the operation room, her baseline vital signs were as follows: heart rate (HR), 82 bpm with normal sinus rhythm; noninvasive blood pressure (BP), 130/76 mmHg; and oxygen saturation (SpO_2_), 96%. Supplemental oxygen was administrated by a face mask at the rate of 3 L/min. Traditional epidural puncture was performed at the L_1–2_ and L_3–4_ interspaces in the left lateral position, and a catheter was inserted to a depth of 4 cm into the epidural space at the cranial and caudal ends of the surgical field. The patient was then placed supine in a left-tilt position; 3 mL of 2% lidocaine was injected as a test dose, and another 7 mL was injected 5 min later through the cranial catheter. Additionally, 3 mL of 1.5% lidocaine was injected through the caudal catheter. At 20 min after the lidocaine injection, the sensory block had reached T_6_. The patient had no discomfort, and her hemodynamic parameters were stable. The operating bed was adjusted from the left-tilt to horizontal position, and the operation was allowed. Two minutes later, the patient reported chest distress and difficulty breathing (HR, 110 bpm; BP, 80/69 mmHg; SpO_2_, 96%), and 3 mg of intravenous ephedrine was promptly administered. The patient immediately lost consciousness with no response (HR and BP were depressed to 40 bpm and 53/15 mmHg, respectively). The lowest BP at the time was not measured because the noninvasive BP measurement interval was set to 1 min. The obstetricians sped up the surgery, and in another 2 min, a 2750-g male newborn was delivered with an Apgar score of 10 at both 1 and 5 min. Spontaneous circulation of the patient was returned immediately after delivery, and stable vital signs returned with no other treatment. The postoperative course was uneventful. The mother and neonate recovered uneventfully and were discharged 4 days later.

## Discussion and conclusions

Supine positioning of a pregnant patient will result in aortocaval compression (ACC). Supine hypotensive syndrome may become a life-threatening condition [3], especially for patients with SAS, as demonstrated in our case. Our patient was placed supine in a left-tilt position after epidural anesthesia. She had no discomfort, and her hemodynamic parameters were stable while in a 15° left lateral tilt. Two minutes after the operating bed was moved from the left-tilt to horizontal position, the patient developed sudden loss of consciousness and severe bradycardia and hypotension. We determined that the underlying cause of this severe emergency condition was ACC, which caused a dramatic decrease of venous return. If treated improperly, this emergency condition may rapidly result in maternal cardiac arrest. Under this condition we should not to delay delivery of the newborn because of the mother’s resuscitation. On the contrary, the delivery of the fetus is important in relieving ACC completely, and after delivery, our patient’s spontaneous circulation immediately returned. Therefore, maintaining LUD and relieving ACC are crucial for patients with SAS.

Current clinical practices and guidelines suggest a tilt range of 12 to 15° should be performed for patient undergoing elective CS under neuraxial blockade. In clinical practice, obstetricians often prefer tend to apply a lesser degree of left tilt or even supine position because of subjective feeling of insecurity’ for parturients and very less convenient to perform CS. Although the cardiac output (CO) between with and without tilt at CS has no significant difference in noncardiac patient [4–6], and not lead to deadly outcomes for women with normal pregnancy, but may result in devastating outcomes for pregnancy women with LVOT obstruction, as demonstrated the patient with SAS in our case. Failure to strictly enforce the LUD may be the main cause of sudden loss of consciousness and tend to cardiac arrest in our patient. For those patients, we strongly recommend tilt position should be strictly enforced to relieve ACC from administration of epidural anesthesia to delivery of the fetus. If the obstetricians prefer that the bed is not tilted, high-quality continue manual LUD may be another good choice if technically feasible.

Successful resuscitation occurred in our case because rapid delivery was ensured once the sudden loss of consciousness and severe bradycardia and hypotension were detected. If patients with SAS develop these life-threatening conditions, the anesthesiologist and obstetrician must consider the possibility of severe ACC by the gravid uterus. Delivery of the fetus is critical and should be rapidly initiated.

The two main purposes of timely cesarean delivery are as follows. The first is resuscitation of maternal circulation [2]. Complete relief of ACC by emptying the uterus results in a significant increase in venous return, and cardiac output is then effectively established. The birth is critical to maximize the chance of maternal survival. Secondly, timely delivery is accomplished with a decreased risk of permanent neurological damage from anoxia. When the mother develops sudden loss of consciousness and severe bradycardia and hypotension, timely delivery of the baby may be the best way to optimize the condition of both the mother and fetus. A review showed that timely birth of the fetus after maternal arrest resulted in a clear maternal survival benefit in 19 of 60 cases (31.7%). There were no cases in which delivery of the baby may have been deleterious to maternal survival [7]. Therefore, in patients with SAS, delivery of the fetus should be rapidly initiated once this life-threatening condition is detected.

In conclusion, supine positioning resulted in a life-threatening condition due to ACC in pregnant women with SAS. We strongly recommend the performance of tilt position strictly or high-quality continue manual LUD until the fetus is delivered. If patients with SAS develop sudden loss of consciousness and severe bradycardia and hypotension, timely delivery of the fetus in order to optimize the condition of both the mother and fetus.

## Data Availability

All data related to this case report are contained within the manuscript.

## Abbreviations

ACC: Aortocaval compression
BP: Blood pressure
CHD: Congenital heart diseases
CO: Cardiac output
CS: Cesarean section
HR: Heart rate
LUD: Left uterine displacement
LVOT: Left ventricular outflow tract
PMCD: Perimortem cesarean delivery
SAS: Subvalvular aortic stenosis
SpO_2_: Oxygen saturation
